# Oncoproteins E6 and E7 upregulate topoisomerase I to activate the cGAS-PD-L1 pathway in cervical cancer development

**DOI:** 10.3389/fphar.2024.1450875

**Published:** 2024-08-02

**Authors:** Ying Luo, Mengda Niu, Yanfei Liu, Miaochang Zhang, Yuanyuan Deng, Dan Mu, Junfen Xu, Shiyuan Hong

**Affiliations:** ^1^ College of Pharmacy, Chongqing Medical University, Chongqing, China; ^2^ Department of Gynecologic Oncology, Women’s Hospital, Zhejiang University School of Medicine, Hangzhou, Zhejiang, China

**Keywords:** DDR, TOP1, cGAS, PD-L1, human papillomavirus, cervical cancer

## Abstract

**Background:** Cervical cancer (CC) stands as a significant health threat to women globally, with high-risk human papillomaviruses as major etiologic agents. The DNA damage repair (DDR) protein topoisomerase I (TOP1) has been linked to various cancers, yet its distinct roles and mechanisms in CC are not fully elucidated.

**Methods:** We investigated TOP1 expression in cervical intraepithelial neoplasia (CIN) and CC tissues utilizing qRT-PCR and IHC, correlating findings with patient prognosis. Subsequent knockdown studies were performed *in vitro* and *in vivo* to evaluate the influence of TOP1 on tumor growth, DNA repair, and inflammatory responses.

**Results:** TOP1 was highly expressed in CIN and CC, negatively correlating with patient prognosis. Inhibition of TOP1 impeded CC cell growth and disrupted DNA repair. TOP1 was shown to regulate tumor-promoting inflammation and programmed death-ligand 1 (PD-L1) production in a cGAS-dependent manner. HPV oncoproteins E6 and E7 upregulated TOP1 and activated the cGAS-PD-L1 pathway.

**Conclusions:** TOP1 acts as a DNA repair mediator, promoting CC development and immune evasion. Targeting the TOP1-cGAS-PD-L1 axis could be a potential therapeutic strategy for CC.

## 1 Introduction

Cervical cancer (CC) is a prevalent malignancy, ranking as the fourth most lethal cancer among women worldwide. In 2020, an estimated 604,000 new cases of CC and 342,000 deaths were reported globally (Sung et al., 2021). Despite advancements in therapeutic approaches, the survival rate for patients with recurrent or metastatic CC has not significantly improved. The pathogenesis of CC has been studied for decades, with high-risk human papillomaviruses (HPVs) identified as the major causative agents. However, the underlying mechanisms are not fully elucidated. HPV oncoproteins E6 and E7 are instrumental in the transformation of normal cells into tumor cells through various mechanisms, including p53 degradation (Scheffner et al., 1990) and Rb-dependent E2F activation (Dyson et al., 1989; Chellappan et al., 1992), which trigger molecular events such as apoptosis (Finzer et al., 2002) and DNA damage repair (DDR) (Bruyere et al., 2023). DDR plays a pivotal role in the regulation of tumorigenesis and progression (Wieringa et al., 2016; Su et al., 2018; Ni et al., 2021). Key DDR kinases, such as ataxia telangiectasia mutated (ATM) and ATM and Rad3-related (ATR), are activated by E6 or E7 (Moody and Laimins, 2009; Hong et al., 2015). Inhibition of ATM/ATR induces senescence-related inflammation (Kang et al., 2015). Despite these findings, the comprehensive role of DDR in CC remains poorly understood.

Topoisomerase I (TOP1) is a crucial enzyme in DDR, responsible for relaxing both positive and negative supercoiled DNA during replication or transcription (Pommier et al., 2016). Abnormal expression or mutation of TOP1 has been reported in various solid tumors, including breast cancer (Kümler et al., 2015), metastatic colorectal cancer (Jensen et al., 2016; Palshof et al., 2017), and ovarian carcinoma (Heestand et al., 2017). However, the specific functions and mechanisms of TOP1 in CC require further clarification. TOP1 inhibition by Topotecan (TPT) or Camptothecin (CPT) has shown anti-tumor effects in xenograft models and clinical trial patients (Du et al., 2018; Tsuchihashi et al., 2020; Spinazzi et al., 2022). The poisoned TOP1 is trapped within the TOP1 cleavage complex (TOP1cc), resulting in DNA double-stranded breaks (DSBs) (Pommier et al., 2016) and genomic instability (Pommier et al., 2022). Consequently, TOP1cc formation induces changes in the expression of several homologous recombination (HR) components such as Rad50 (Sacho and Maizels, 2011), BRCA1 (Murai et al., 2012), and BRCA2 (Marzi et al., 2019).

Emerging studies have unveiled additional functions of TOP1 (Onishi and Kawano, 2012) beyond its involvement in DDR. TOP1cc has been found localized in cytoplasmic chromatin, along with cyclic GMP-AMP synthase (cGAS), which can sense increased intrinsic DNA breaks and trigger downstream inflammatory cascades (Zhao et al., 2020). Consistently, TOP1 inhibition regulates the production of pro-inflammatory cytokines and chemokines during viral infection (Rialdi et al., 2016; Riedlinger et al., 2019; Ho et al., 2021). Upon activation, cGAS generates a second messenger called 2′3′-cyclic-GMP-AMP (cGAMP), which binds to the stimulator of interferon genes (STING) and triggers two distinct signaling pathways. One pathway involves TANK-binding kinase 1 (TBK1)-dependent phosphorylation of interferon regulatory factor 3 (IRF3), leading to the production of type I interferons (IFNs), inflammatory mediators, and interferon-stimulated genes (ISGs) (Ishikawa et al., 2009; Sun et al., 2013). The alternative pathway involves the activation of nuclear factor kappa B (NF-κB) signaling, known to foster tumor cell growth (Huber et al., 2004). Furthermore, the cGAS-STING pathway’s activation is linked to the induction of programmed death ligand 1 (PD-L1) and recruitment of T-cells in small-cell lung cancer (Sen et al., 2019). PD-L1 binds to the PD-1 receptor and inhibits T-cell proliferation, allowing cancer cells to evade immune surveillance of the host (Yang et al., 2018). Although alterations in PD-L1 protein levels have been observed in advanced cervical cancer (Loharamtaweethong et al., 2020), it remains unclear whether TOP1 regulates PD-L1 expression during CC progression. Expression of cGAS is induced by HPV E6 (Gusho and Laimins, 2022), but it has been suggested to be involved in cell death sensitization. The potential regulation of cGAS by TOP1 in promoting CC tumorigenesis warrants further investigation.

Our study presents evidence that elevated TOP1 expression correlates with poor prognosis in CC patients. Both *in vitro* cellular assays and *in vivo* xenograft mouse models have collectively illustrated that TOP1 promotes CC growth and triggers DNA repair. Notably, TOP1 regulated the PD-L1 production in a cGAS-dependent manner. Depletion of TOP1 suppressed the expression of cGAS, NF-κB, and PD-L1, without impacting IFN-β. Moreover, compensation of cGAMP could restore PD-L1 levels in TOP1-depleted cells. In addition, oncoproteins E6 or E7 not only upregulated TOP1 levels but also interacted with cGAS or TOP1, thereby promoting PD-L1 protein accumulation. Our analyses also identified other downstream targets of TOP1, encompassing certain cytokines, chemokines and T cell regulatory genes. Taken together, our work revealed the significance of TOP1-cGAS-PD-L1 signaling axis in CC development, providing a promising avenue for developing novel therapeutic strategies against CC.

## 2 Materials and methods

### 2.1 Clinical tissue samples

A total of 63 samples, including primary CC, cervical intraepithelial neoplasia (CIN), and adjacent normal tissues, were obtained with informed consent from the Women’s Hospital School of Medicine, Zhejiang University Review Board (Zhejiang Province, China). All tumor specimens were pathologically diagnosed, and non-tumor specimens were verified according to standard procedures. None of the patients included in this study had undergone pre-operative adjuvant chemotherapy or radiation therapy. The clinical characteristics of all CC patients are listed in Supplementary Table S1.

### 2.2 Cell culture and chemical reagents

Human CC cell lines SiHa, CaSki, and HeLa were purchased from Procell Life Science and Technology Co., Ltd. (Wuhan, China). HaCaT, HEK-293T, and PT67 cells were obtained from the American Type Culture Collection (ATCC). SiHa and HaCaT cells were cultured in MEM medium (Gibco). CaSki cells were cultured in RPMI-1640 medium (Gibco). HEK-293T, HeLa, and PT67 cells were cultured in DMEM medium (Gibco). All culture media were supplemented with 10% fetal bovine serum (FBS, Adamas), streptomycin (100 μg/mL), penicillin (100 units/mL), and Amphotericin B solution (0.25 μg/mL). Antibodies, chemical reagents, and experimental kits are listed in Supplementary Tables S2, S3.

### 2.3 RNA extraction and quantitative real-time PCR analysis (qRT-PCR)

Total RNA was isolated from cultured cells using TRIzol reagent (Invitrogen, cat# 15596026) following the manufacturer’s instructions. RNA concentration and quality were assessed using the NanoDrop 2000 spectrophotometer (Thermo Scientific). RNA was reverse transcribed into cDNA using the RT Master Mix (MCE, HY-K0511A) in a thermal cycler (ETC811). Real-time PCR was performed using SYBR Green qPCR Master Mix (MCE, HY-K0501A) on the QuantStudio™ 1 Real-Time PCR Instrument (Bio-Rad Laboratories, United States). Relative expression levels of target genes were calculated using the comparative CT (2^−ΔΔCT^) method and normalized to β-actin or GAPDH levels. The qPCR assays were carried out in triplicate biological replicates and statistical significance was determined using Student’s t-test, with a significance level set at *P* <0.05 was considered significant. Human qPCR primer sequences are listed in Supplementary Table S4 and were available from the online PrimerBank database (https://pga.mgh.harvard.edu/primerbank/).

### 2.4 Preparation of protein lysates and Western blot

For the preparation of total protein lysates, cervical cancer cells were washed twice with ice-cold phosphate-buffered saline (PBS) and subsequently lysed on ice using RIPA Lysis buffer (Solarbio, R0010), supplemented with a protease and phosphatase inhibitor cocktail (MCE, HY-K0013). Cell lysates were then incubated on ice for 30 min with occasional vortexing. After removing precipitates, the protein concentration of the clean supernatants was quantified using the BCA protein assay kit (Beyotime, P0010S).

Equal amounts of protein lysates (30 μg) were separated by SDS-PAGE, and the gels were transferred to PVDF membranes using the BioRad system. Blocking of non-specific binding was performed by incubating the membranes in 5% nonfat milk at room temperature (RT) for 1 h. The primary antibodies were then incubated with the membranes overnight at 4°C. Goat anti-rabbit or mouse horseradish peroxidase (HRP)-labeled secondary antibodies were further incubated with the membranes for 1 h at RT. Chemiluminescent signals were detected using the Image QuantTM LAS 500 instrument. The antibody resources and dilutions used in this study are listed in Supplementary Table S2.

### 2.5 Lentiviral transduction and establishment of TOP1 knockdown cell lines

The sh-TOP1 plasmids used in our study were designed and synthesized by GeneChem Co., Ltd. (Shanghai, China). The 293T cells were transfected with TOP1-specific shRNAs (sh-TOP1) and scrambled shRNA (sh-NC) using Lipofectamine 3000 (Invitrogen), following the manufacturer’s protocol. The viral particle supernatant generated was collected and used to transduce SiHa and HeLa cells at optimized concentrations. The transduced cells were selected using puromycin. The efficacy of knockdown was subsequently verified through both qRT-PCR and Western blot analyses.

### 2.6 Generation of HPV E6/E7 knockdown or overexpression cells

The shRNAs targeting HPV16 E6/E7 and HPV18 E6/E7 were purchased from Tsingke Biotechnology (Shanghai, China). Sequences of these plasmids are listed in Supplementary Table S5. HeLa and SiHa cells were transfected with E6 or E7 shRNA or scrambled sequences using Lipofectamine 3000 reagents according to the manufacturer’s protocol. Subsequently, the efficiency of knockdown cells was verified using Western blot.

The pLXSN vector, 16E6, 16E7, 18E6, and 18E7 were generously provided by Dr. Laimonis A. Laimins (Northwestern University, Chicago, United States) and Dr. Frank Stubenrauch (University of Tübingen, Tübingen, Germany). The specific retroviral constructs targeting E6 or E7 were applied as previously described (Halbert et al., 1991; Hebner et al., 2007). The individual viral plasmid was transfected into PT67 packaging cells to generate viral particles. HaCaT cells were transduced with viral supernatants and screened using G418 to establish stable cells expressing HPV16/18 E6 or E7. The efficiency of overexpression cells was verified using Western blot.

### 2.7 Cell counting Kit-8 (CCK8) assay

Cells were seeded in 96-well plates at a density of 5,000 cells per well and cultured for 0–120 h. Subsequently, 10 μL of CCK8 (MCE, HY-K0301) was added to each well and incubated for 1 h at 37°C. The absorbance of cells at each time point was determined using a microplate reader (Thermometer Varioskan LUX) at a wavelength of 450 nm. Cell viability was analyzed using GraphPad Prism 8 software. Each group was performed in three replicates and repeated in three independent biological experiments.

### 2.8 Wound healing assay

Cells were cultured in 6-well plates until they reached 90%–100% confluence. The cell monolayers were vertically scratched in the center of the well using a 200 μL micropipette tip. After washing away the floating cells with PBS, the remaining attached cells were incubated in a serum-free medium for 24 h at 37°C. Representative images were captured at 0 h and 24 h after the injury using a microscopy imaging system. The width of the baseline and wound healing region was measured using ImageJ. The cell migration rate was calculated as 100% × (width of a wound at 0 h - width of a wound at 24 h)/width of a wound at 0 h. The experiment was independently repeated in triplicates.

### 2.9 Transwell assay

A total of 30,000 cells were seeded into the upper chamber of a 24-well plate (Corning, United States) with serum-free medium (200 μL), while the lower chamber was filled with fresh medium (500 μL) containing 20% FBS. After 24 h of incubation, cells in the upper chambers were fixed using 4% PFA and subsequently stained with crystal violet. For the cell invasion assay, the upper chamber was initially coated with Matrigel (BD Bioscience, 356234) in the serum-free medium before the cells were seeded for subsequent procedures. Representative fields were captured at ×100 magnification using an OLYMPUS BX53 microscope. The number of migrated or invaded cells was calculated using ImageJ.

### 2.10 Real-time label-free cell analysis technique (RTCA)

The E-Plate 96 was prepared by adding 50 μL of medium to each well, followed by placing it in the RTCA Station for plate scanning and initiating Step 1 for baseline detection. A cell suspension with a concentration of 5,000 cells/100 μL was prepared and added to the E-Plate to ensure thorough mixing with the medium from the first step. After incubating the plate for 30 min at room temperature, the E-Plate 96 was returned to the RTCA Station incubator, and the system was initiated to automatically scan the plate. The parameters of the RTCA program included cell type (SiHa, HeLa), cell number (5,000), interval (10 min), unit (min), total time (120 h), and the name of the compound (sh-NC, sh-TOP1#2, sh-TOP1#3). Subsequently, Step 2 was initiated to detect the cell proliferation curve.

### 2.11 Colony formation assay

A total of 800 cells were seeded in 6-well plates. Cells were observed during the incubation process until the majority of individual clones contained more than 50 cells, indicating completion of the cloning process. This typically took about 10–14 days. Following incubation, the supernatant of the cell culture was discarded, and the cells were washed with PBS and fixed with 4% PFA at RT for 30 min. After fixation, cells were washed again with PBS, and a crystal violet stain solution was applied to each well, staining at room temperature for 20 min. Following staining, cells were washed multiple times with PBS. Once air-dried, the 6-well plates were photographed using a digital camera. The individual clones were then quantified based on the captured photographs.

### 2.12 EdU-incorporation assay

Proliferation ability was assessed using the EdU incorporation assay following the manufacturer’s protocol of EdU Imaging Kits (Cy3) (APExBIO, K1075). Cells in logarithmic growth phase were inoculated and cultured overnight, then divided into DMSO group and inhibitors (G140, G150) treatment group respectively. The inhibitor was used at a final concentration of 5 µM for 24 h. After inhibitor treatment, the drugs were removed, and cells were treated with a completely diluted EdU working solution in the culture medium, with a final concentration of 10 µM. Cells were incubated with EdU for 2 h at 37°C. Then cells were washed twice and fixed with 4% paraformaldehyde at room temperature for 15 min 0.3% TritonX-100 was added to the cells and incubated at room temperature for 15 min after washing with PBS. After cell permeability was achieved, the Click reaction solution was added to the cells and incubated at RT in the dark for 30 min. Finally, Hoechst33342 was added to the cells for nuclear staining in the dark for 15 min at RT. Image analysis was performed using a confocal microscope to observe and analyze the EdU-labeled and unlabeled cells.

### 2.13 Co-immunoprecipitation (Co-IP)

Cells were lysed on ice for 30 min using IP lysis buffer (Beyotime, P0013) supplemented with protease and phosphatase inhibitor cocktails. Lysates were then quantified for protein concentration using the BCA Protein Assay Kit. Subsequently, the total lysates were divided into three groups: the input control group (50 μL lysates processed with loading buffer), the IP group, and the IgG group. The latter two groups were incubated with specific antibodies according to the manufacturer’s instructions overnight. Following this, 20 μL of protein A&G sepharose beads (Beyotime, P2055) were added to the mixture and rotated for 2 h at 4°C. The beads of the mixture were washed three times with the IP lysis buffer, and the immunoprecipitated protein complexes were resuspended in 2× SDS-PAGE protein sample loading buffer (Beyotime, P0288) for further analysis by Western blot.

### 2.14 Immunofluorescence (IF)

Cells were seeded in 35 mm glass-bottom dishes (Ibidi), then fixed with 4% PFA (Biosharp, BL539A) for 15 min, permeabilized in PBS containing 0.1% Triton X-100 for 15 min, and blocked for 1 h at room temperature. They were then incubated with primary antibodies overnight at 4°C (TOP1 1:200, cGAS 1:200) or (BRCA1 1:200, γH2AX 1:500). The following day, cells were washed three times with PBS and incubated with secondary antibodies (488 donkey anti-Mouse 1:2000, 555 donkey anti-Rabbit 1:500) in the dark for 1 h. After further washes, the cells were mounted with DAPI-containing mounting medium (Solarbio, S2110), and visualized using a laser scanning confocal microscope (Nikon A1R). Subsequent image processing was performed using NIS-Elements Viewer 4.20 software, and colocalization analysis with endosome markers was conducted using Fiji/ImageJ.

### 2.15 Comet assay

Comet slides were pre-coated with cells (avoiding light), mixed with molten low melting agarose (LMA) at a ratio of 1:10, and then placed in lysis solution at 4°C overnight. Slides were further incubated in a cold neutral electrophoresis buffer for 30 min followed by electrophoresis for 45 min at 21 V using a Comet Assay^®^ ES unit. Slides were treated with DNA precipitation solution followed by a wash with 70% ethanol. The dried slides were stained with SYBR Gold dye (1:10000, Invitrogen, S11494) in the dark and visualized using an OLYMPUS BX53 microscope at an excitation/emission wavelength of 496/522 nm. The comet tail length was quantified using CASP software analysis.

### 2.16 Xenograft formation assay

Female BALB/c nude mice (4–5 weeks old) were procured from Changzhou Cavens Laboratory Animal Co., Ltd. (Changzhou, Jiangsu, China) and housed under specific pathogen-free (SPF) conditions, with a temperature of 25°C, 50% humidity, and a 12/12 light/dark cycle. All mouse experiments adhered to the standard guidelines of the Institutional Animal Care and Use Committee of Chongqing Medical University. After random division into sh-NC and sh-TOP1 groups (n = 5/group), the mice received subcutaneous injections of CC cells (2 × 10^6^) suspended in a 1:1 mixture of media and Matrigel (BD Biosciences). Tumor length (L), width (W), and weight were measured using a caliper and an electronic scale. Tumor volumes (mm3) were calculated using the formula: 0.5 × L × W^2^.

### 2.17 Immunohistochemical (IHC) and Hematoxylin and eosin (HE) staining

Tumor tissues were fixed in 4% paraformaldehyde, dehydrated in alcohol with gradient percentages, and embedded in paraffin. Paraffin sections (5 μm) were cut into slides, dewaxed in xylene, hydrated, and blocked for endogenous peroxidase activity using Beyotime’s P0100B solution. Slides underwent antigen retrieval (pH 9.0) using heat-induced EDTA solution (Solarbio, C1034), followed by incubation in blocking buffer (Beyotime, P0260) at 37°C in a humidified chamber. Subsequently, slides were incubated with diluted primary antibodies at 4°C overnight, followed by a secondary antibody conjugated to HRP (zsbio, PV-6000). DAB substrate solution (zsbio, PV-8000) was applied to visualize antibody staining. Hematoxylin was used for counterstaining, followed by dehydration with ethyl alcohol, clearing with xylene, and mounting with neutral resin. Images of antibody staining were captured using an OLYMPUS BX53 microscope.

For HE staining, tissue sections were dewaxed and hydrated before staining the nucleus with Hematoxylin Solution (Solarbio, G1120) for approximately 90 s. Slides were then washed with water and stained with Eosin Y Aqueous Solution for 10–15 s. Slides were dehydrated with dimethylbenzene and mounted with resinous medium in a routine order.

### 2.18 Measurement of IFNβ and 2′3′-cGAMP production

To quantify serum IFNβ proteins, blood samples from tumor-bearing mice were collected into non-heparinized tubes, left to clot at room temperature for 30 min, and then centrifuged for 15 min at 1,000 g to collect serum. Serum levels of IFNβ protein were detected using the Human IFNβ ELISA Kit (Proteintech, KE00187). IFNβ protein was captured by IFNβ antibody pre-coated onto microwells. After extensive washing, wells were incubated with a second biotinylated antibody. Subsequently, streptavidin-HRP reagent and tetramethyl-benzidine substrate (TMB) were added for color development, and the reaction was stopped with sulfuric acid before measuring absorbance at 450 nm using a microplate reader (Thermometer Varioskan LUX).

For the measurement of 2′3′-cGAMP production, cell lysates of the TOP1 knockdown group and control group were prepared and quantified using the 2′3′-cGAMP ELISA kit according to the manufacturer’s instructions (Cayman, 501700). Concentration of 2′3′-cGAMP in cell samples was calculated based on standard curves.

### 2.19 Flow cytometry-based cell cycle and apoptosis analysis

For cell cycle analysis, cells in the logarithmic phase were seeded at 1 × 106 cells/mL in 6-well plates, with 2 mL of medium per well. After a specified culture duration, the cells were harvested by centrifugation at 800 rpm for 5 min, followed by washing with cold PBS. The pellet was fixed in cold 75% ethanol for over 4 h at 4°C. The fixed cells were then centrifuged again, washed with PBS, and resuspended in a staining solution containing propidium iodide (PI, 50 μg/mL) and RNase A (100 μg/mL). After incubation at 4°C in the dark for 60 min, the cells were analyzed using a flow cytometer, with 20,000 to 30,000 cells counted per sample. Data analysis and cell cycle profiling were conducted using specialized software.

For apoptosis detection, adherent cells were cultured and the medium was transferred to centrifuge tubes after washing with PBS. Following trypsinization with 1 mL of EDTA-free trypsin and neutralization with 3 mL of serum-containing medium, cells were centrifuged at 1,000 rpm for 5 min. The pellet was resuspended in PBS and centrifuged again before being resuspended in 1–2 mL of Annexin V Binding Buffer (AVBB). To this, 100 μL of PI staining mixture and 2–4 μL of Annexin V-FITC were added, and the cells were incubated at room temperature in the dark for 15 min. After centrifugation and removal of the supernatant, the cells are resuspended in 500 μL of AVBB and kept on ice until flow cytometry analysis.

### 2.20 Online database analysis

Gene Expression Profiling Interaction Analysis, GEPIA (Tang et al., 2017) (http://gepia2.cancer-pku.cn /#index) was employed to assess the relative expression levels of TOP1 in human CC and control tissues, as well as to investigate the association of TOP1 expression with CC survival. Tumor Immune Estimation Resource, TIMER2.0 (Li et al., 2020) (http://timer.cistrome.org/) was utilized to analyze the correlation between TOP1 and defined DDR genes (FANCD2,BRCA1,RAD51) and cGAS in CC. GeneMANIA (Franz et al., 2018) (http://genemania.org/) was used to explore gene list, analyze interactions and functions between TOP1 and target genes. The Assistant for Clinical Bioinformatics (https://www.aclbi.com/) is an online tool provided by HOME for Researchers (https://www.home-for-researchers.com/). This tool analyzes the correlation between gene and pathway scores using Spearman’s rank correlation based on the GSVA package in R software. A *p*-value of less than 0.05 was considered statistically significant.

### 2.21 Statistical analysis

Statistical analyses were conducted using SPSS 22.0 and GraphPad Prism 8.0 software for each experiment. Data were presented as mean ± SD. Statistical differences were assessed by unpaired two-tailed Student’s t-test between two independent groups, one-way ANOVA test, or Newman Keul’s multiple comparison test for comparisons involving more than two groups. Spearman’s rank-order correlation test was employed for correlation analysis. A *p*-value less than 0.05 was considered statistically significant, denoted as * (*P* < 0.05), ** (*P* < 0.01), *** (*P* < 0.001), while “NS” indicates no significance. The Western blot, qRT-PCR, Co-IP, IHC staining analyses, along with animal experiments were conducted using biological replicates as indicated in the Figure legend and corresponding methods section.

## 3 Results

### 3.1 TOP1 expression is upregulated in CC and correlated with poor prognosis

**FIGURE 1:**
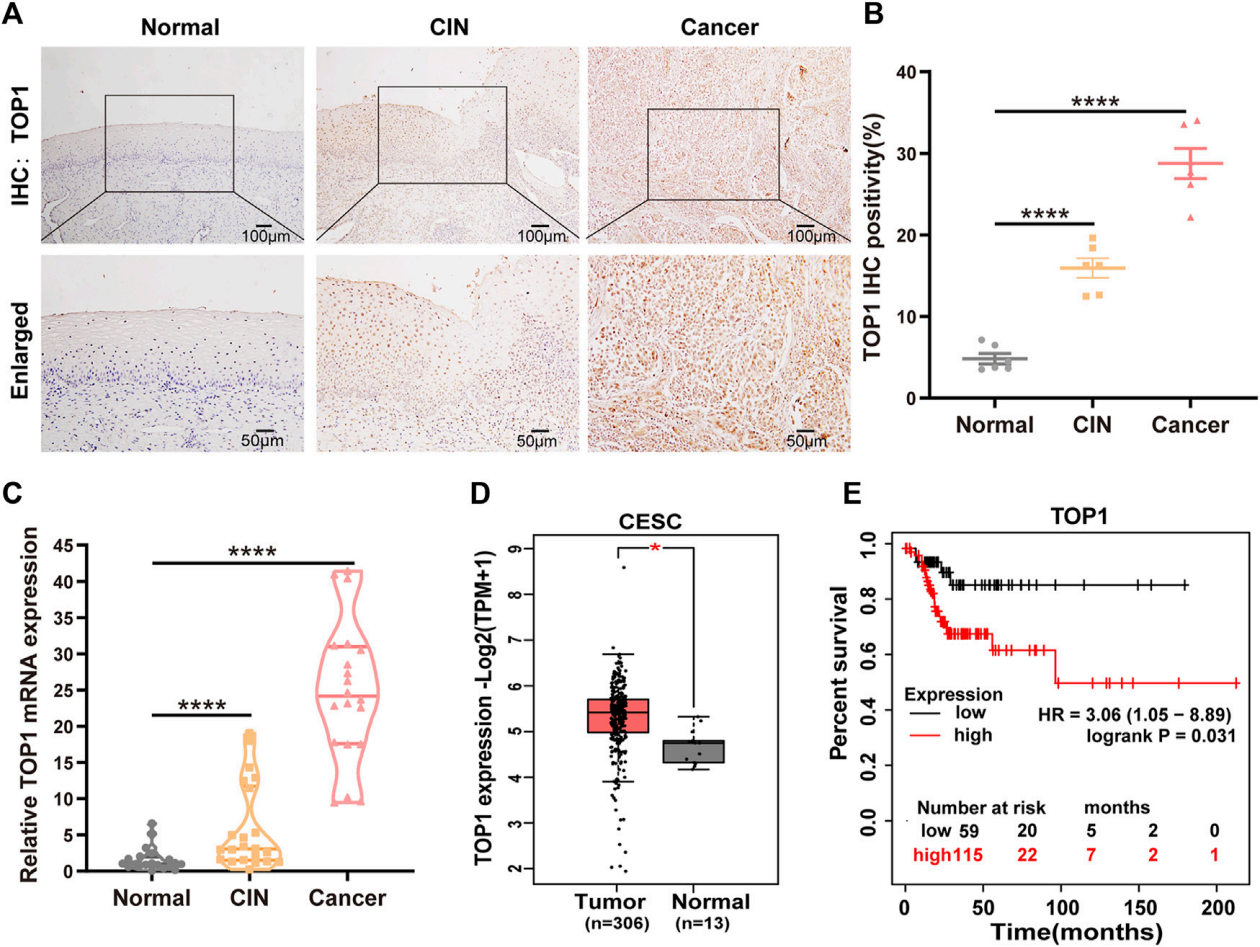
High expression of TOP1 is associated with pathological grades and poor prognosis of CC. **(A)** Representative immunohistochemical staining images of TOP1 were observed in cervical tissues at different stages. Scale bar = 50–100 μm. **(B)** Quantitative analysis of expression of TOP1 based on the percentage of positively stained cells. **(C)** The relative mRNA expression levels of TOP1 were detected using qRT-PCR among normal group (n = 21), CIN group (n = 22), and CC group (n = 20). The qRT-PCR was conducted at least in triplicates. Significant differences by one-way ANOVA test. **(D)** The TOP1 mRNA expression levels in normal tissues (n = 13) and CC tissues (n = 306) were obtained from the GEPIA database. **(E)** The survival curves of CC patients based on TOP1 expression levels were generated by the GEPIA database. All values are expressed as mean ± SD. Significant differences are indicated as follows: ns not significant, *P < 0.05, **P < 0.01, ***P < 0.001 and ****P < 0.0001.

To investigate the expression levels of TOP1 in various stages of CC development, we examined TOP1 expression at the protein and mRNA levels in CC tissues. Utilizing IHC, we observed a significant increase in TOP1 levels in both precancerous lesions and tumors compared to adjacent noncancerous tissues (Figures 1A, B). Similarly, TOP1 mRNA levels were markedly elevated in CIN or tumor tissues compared to adjacent noncancerous tissues (Figure 1C). Consistently, analysis of the GEPIA databases also indicated upregulation of TOP1 mRNA levels in tumor tissues compared to normal controls (Figure 1D). Moreover, survival analysis using the GEPIA database revealed a negative correlation between high TOP1 expression and survival in CC patients (Figure 1E), suggesting that TOP1 may serve as a poor prognostic marker for CC. Taken together, these findings underscore the presence of aberrant TOP1 expression in CC.

### 3.2 Downregulation of TOP1 suppresses CC cell growth and disrupts DNA damage repair

**FIGURE 2:**
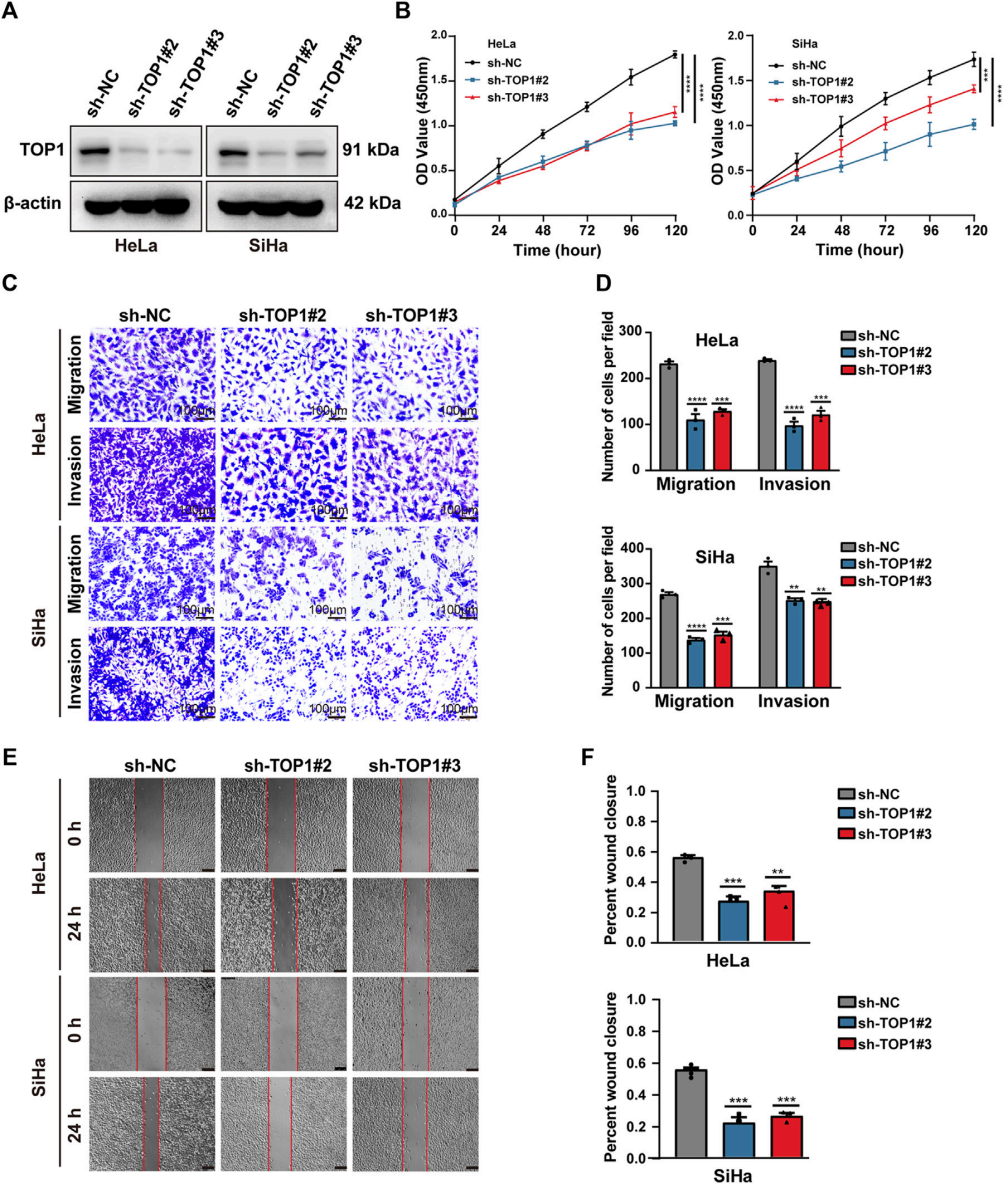
TOP1 knockdown inhibits CC cell proliferation, migration and invasion. **(A)** The knockdown efficiency of TOP1 was measured using Western blotting in SiHa and HeLa cells. **(B)** Cell proliferation of sh-NC or sh-TOP1 cells was assessed using CCK8 assay within 5 days. **(C,D)** Transwell assay showed inhibition of TOP1 suppressed tumor cell migration and invasion pro-cesses. The results were presented in bar graphs for simplicity. Scale bar = 100 μm. **(E,F)** Wound healing assay demonstrated TOP1 knockdown reduced tumor cell migration, illustrated as the bar figures. Scale bar = 100 μm **P < 0.01; ***P < 0.001; ****P < 0.0001.

The expression of TOP1 was validated in CC cell lines, including both HPV-positive cells (CaSki, SiHa, and HeLa) and an HPV-negative cell line (HaCaT), using Western blot analysis. Compared to HaCaT, HPV-positive CC lines exhibited higher levels of TOP1 (Supplementary Figures S1A, S1B). To investigate the role of TOP1 in CC, stable knockdown of TOP1 was achieved in SiHa and HeLa cells using shRNA technology, as confirmed by qRT-PCR (Supplementary Figure S1C) or Western blot analysis (Figure 2A). The generated cells were then subjected to various assays to evaluate cell proliferation, including Cell Count Kit-8 (CCK-8), RTCA, and colony formation. TOP1 knockdown cells exhibited significantly suppressed cell growth rates compared to the control group (Figure 2B; Supplementary Figures S1D–F), indicating that TOP1 inhibition greatly impairs cell survival in CC cells. Furthermore, the transwell assay (Figures 2C, D) revealed a notable decrease in the number of cells migrating to the lower chamber. Consistently, there was a significant reduction in the number of invaded cells in the TOP1-silencing cells. Additionally, the wound healing assay (Figures 2E, F) showed a slower extent of cell migration in the knockdown cells compared to control cells. Collectively, these results clarify that TOP1 promotes CC cell proliferation, migration, and invasion.

**FIGURE 3:**
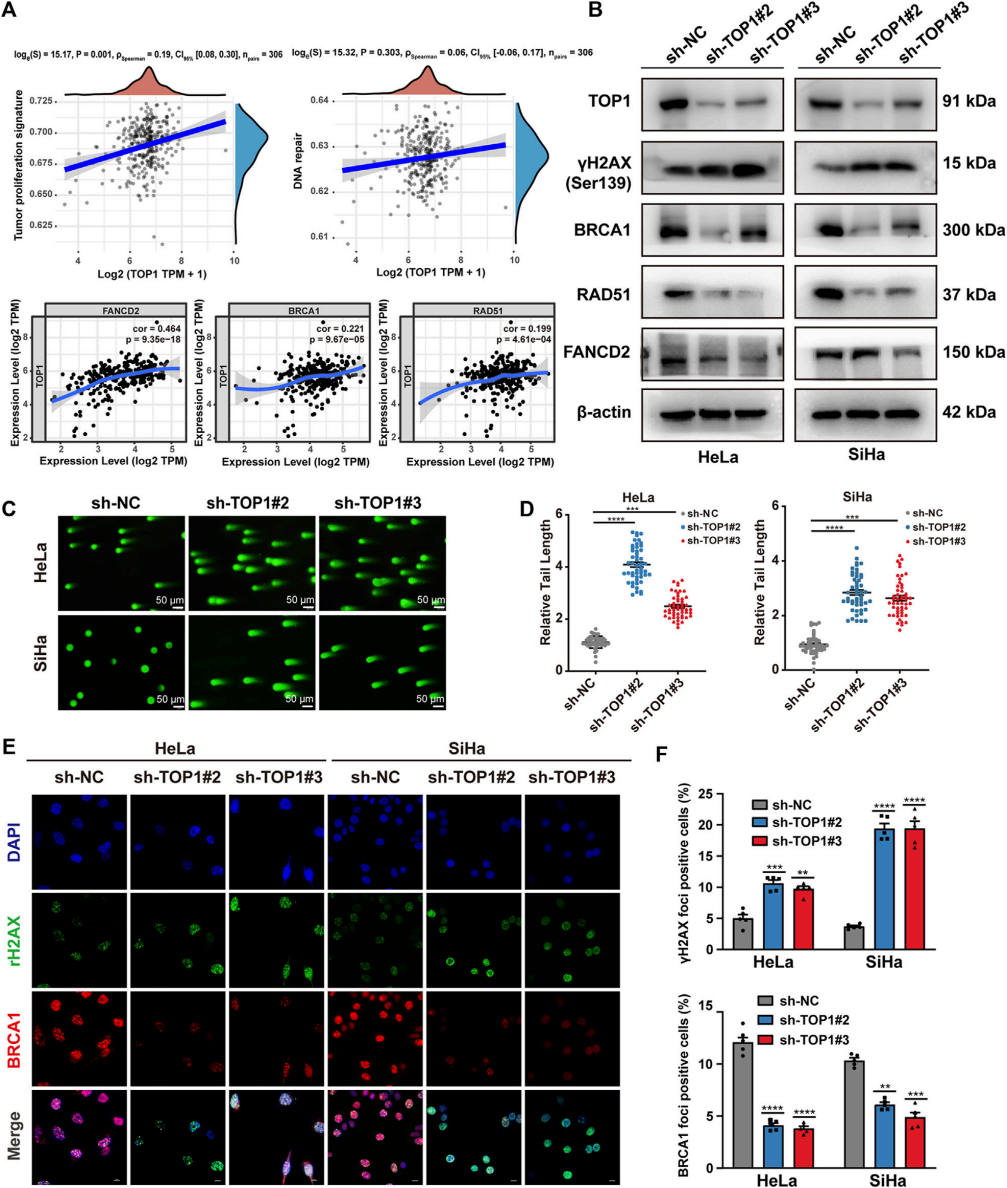
TOP1 depletion significantly inhibits homologous recombination (HR) repair. **(A)** Home for Researchers platform is accessible online for assessing the correlation between individual gene and the specific pathways. The abscissa represents the distribution of the gene expression, and the ordinate represents the distribution of the pathway score. The value on the top represents the correlation *p*-value, correlation coefficient and correlation calculation method. Correlation module from TIMER database draws the expression scatterplots between defined DDR genes and TOP1 in CC, together with the Spearman’s rho value and estimated statistical significance. **(B)** Representative DNA double-strand break (DSB) and HR repair protein expression in the TOP1 knockdown and control CC cells. β-actin was used as endogenous control. **(C,D)** Representative images of the comet assay in TOP1 deficient cells. Scale bar = 50 μm. Quantification of the tail lengths of the comets in CC cells was analyzed using CASP fin three independent experiments. **(E,F)** Immunofluorescence staining of DAPI (blue), γH2AX (Green), and BRCA1 (Red) in TOP1 deficient cells, Scale bar = 10 μm. The percentage of the represent positive cells were quantified from three independent experiments, one-way ANOVA test was used for statistical analysis. **P < 0.01; ***P < 0.001; ****P < 0.0001.

The occurrence of irreversible DNA damage and repair defects often leads to the inhibition of cell growth. The correlations between TOP1 and specific pathways, such as tumor proliferation and DNA repair, were analyzed using the online tool. Analysis of TIMER database further reveals a correlation between TOP1 expression and defined DNA damage repair genes (FANCD2, BRCA1, RAD51) in CC (Figure 3A). The expression of these DDR molecules was subsequently validated using Western blot (Figure 3B). Furthermore, we performed the comet assay to examine whether there is an in-crease in DNA damage in TOP1 knockdown cells (Figure 3C). In this assay, the comet head represents intact genomic DNA, while the tail moment represents DNA fragments generated from DNA breaks. Our fluorescence microscopic data revealed that the comet tail lengths of the TOP1 knockdown cells were significantly longer compared to those in the control cells, suggesting that the loss of TOP1 induces more DNA breaks. The relative tail length was illustrated as bar figures (Figure 3D). Consistent with these results, IF analysis (Figures 3E, F) demonstrated a substantial increase in the number of γH2AX foci and a decrease in BRCA1 foci. γH2AX serves as a marker of double-strand breaks (DSBs), while BRCA1 regulates HR-mediated repair, respectively. The cell cycle distribution in TOP1 knockdown cells (Supplementary Figure S3A, B), indicates a significant G2/M phase arrest, which is consistent with the role of TOP1 in DNA damage repair. This arrest likely serves as a protective mechanism to facilitate the repair of DNA damage. The increase in apoptotic cells (Supplementary Figures S3C, D) underscores the importance of TOP1 in maintaining genomic stability. The absence or reduction of TOP1 activity may lead to unresolved DNA damage and subsequent cell death through the intrinsic apoptotic pathway. Taken together, these findings elucidate that TOP1 deletion effectively impedes DNA repair in CC cells, which suggests that TOP1 acts as a critical factor in CC cell survival.

### 3.3 Downregulation of TOP1 markedly suppresses xenograft tumor growth of CC *in vivo*

**FIGURE 4:**
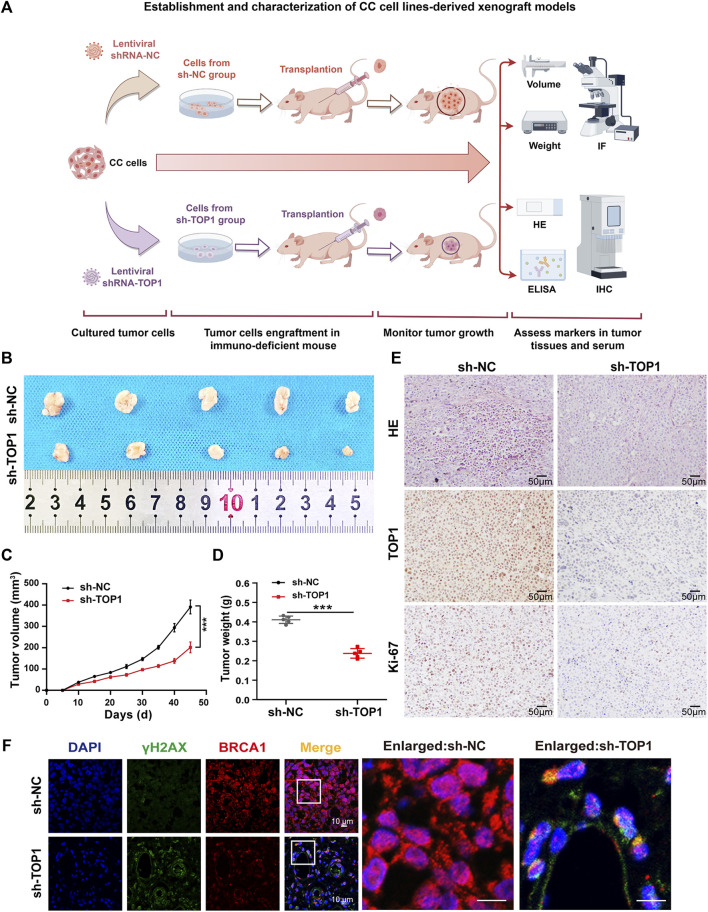
Silencing TOP1 expression suppress the formation of CC *in vivo*. **(A)** Schematic diagram of the establishment of subcutaneous cervical cancer cell-bearing mice. **(B)** The representative endpoint images of the xenograft tumors from BALB/c nude mice injected with 2 × 106 sh-NC and sh-TOP1 CC cells (n = 5 mice/group). **(C,D)** The tumor volume and tumor weight of the mice in two groups were measured every three or 4 days. The growth curves of the xenograft tumors experiments were quantified using GraphPad Prism (n = 5 mice/group). The statistical analysis with one ANOVA test. ***P < 0.001. **(E)** HE staining of the xenograft tumors of euthanized mice and IHC staining of TOP1 and Ki67 proteins. Scale bar = 50 µm. **(F)** Confocal microscopy images of tumor tissues with DNA (blue), γH2AX (green), and BRCA1 (red) staining. Scale bars = 10 μm. All results are representative of observations from three biological replicates.

To further investigate the impact of TOP1 expression on the oncogenic behavior of CC *in vivo*, xenograft tumor models were established using TOP1 scramble control and TOP1 knockdown CC cells (Figure 4A). Identical numbers of CC cells (2 × 10^6^/group) were subcutaneously implanted into BALB/C nude mice. The cell-injected mice were maintained on a normal diet, and the effect of TOP1 inhibition on tumor formation was assessed. The generated tumor was then isolated from the mice for further analysis. Compared with the control group, tumor volumes and weights were markedly decreased in the TOP1 knockdown group (Figures 4B–D). Hematoxylin and eosin (HE) staining showed that the control mice exhibited a tumor phenotype, which was suppressed in the knockdown group. Consistently, the IHC analysis demonstrated that the levels of Ki-67, a well-known marker for tumor cell proliferation, were greatly lower in the knockdown groups. Similarly, TOP1 expression was significantly reduced in the tumor in the knockdown groups (Figure 4E). Moreover, the representative confocal images of tumor tissues demonstrated that the loss of TOP1 induced an increase of γH2AX levels but a decrease of BRCA1 levels (Figure 4F), consistent with the observation in CC cells. Taken together, these findings indicate that TOP1 promotes the formation of CC *in vivo*.

### 3.4 TOP1 increases PD-L1 expression in cGAS-dependent manner

**FIGURE 5:**
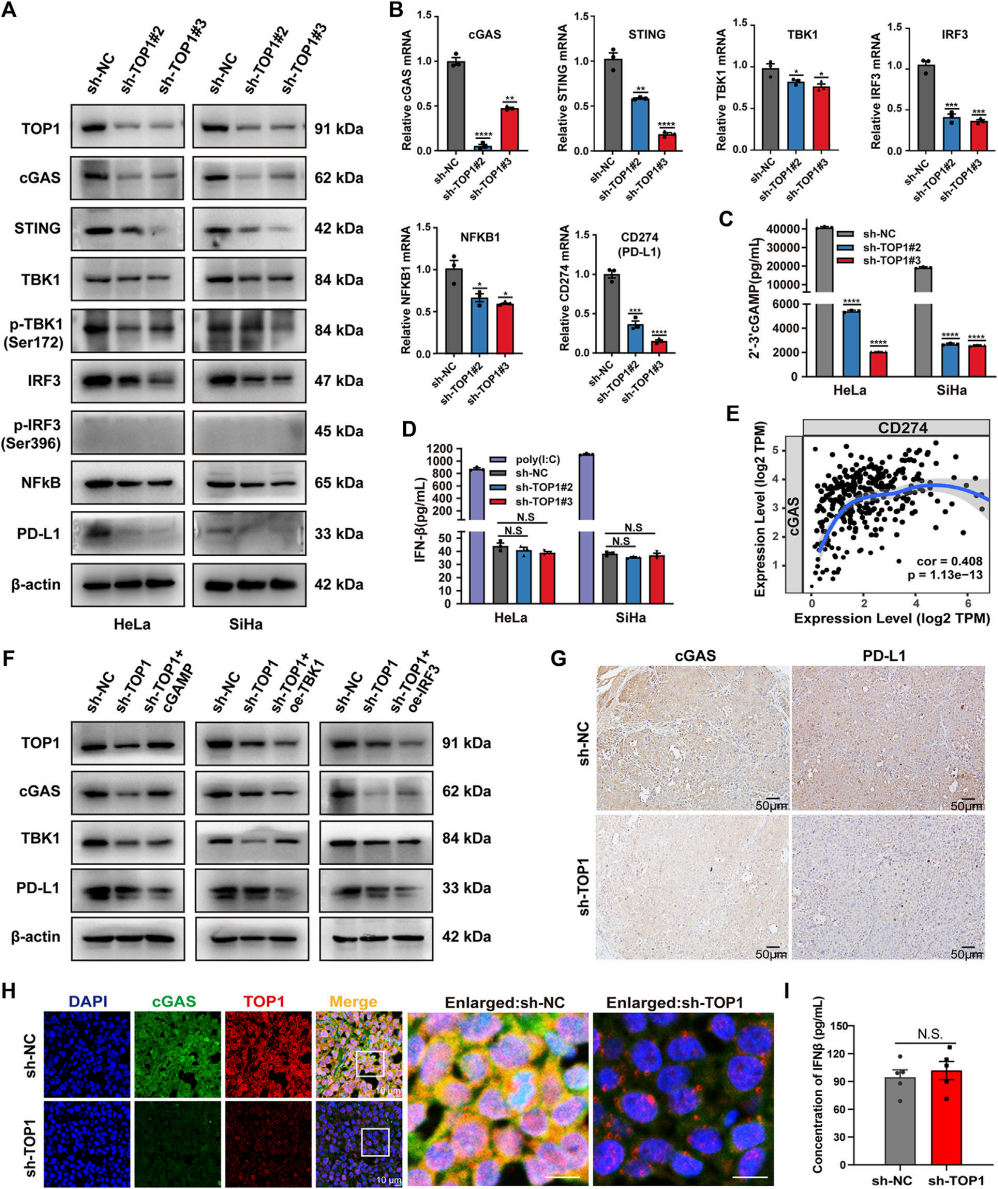
TOP1 is required for activation of the cGAS–PD-L1 pathway in CC. **(A)** Western blot analysis of indicated protein levels of the cGAS pathway in the TOP1 knockdown and control cells. **(B)** qRT-PCR was used to examine the gene expression of TOP1, cGAS, STING, TBK1, IRF3, NF-κB and CD274 (PD-L1) in the TOP1 knockdown and control cells. **(C)** 2′-3′-cGAMP production from CC cell lysate was measured by ELISA assay and normalized by total protein concentration. **(D)** IFNβ (pg/mL) production was measured by ELISA in TOP1 knockdown and control cells. **(E)** The expression scatterplots generated to show the correlation between cGAS and CD274 (PD-L1) in CC, with the Spearman’s rho value (Cor = 0.408) and estimated statistical significance (*P* = 1.13e-13). **(F)** Western blot analysis of indicated protein levels in sh-TOP1 cells treated with cGAMP or transfected with HA-IRF3 and HA-TBK1. **(G)** The IHC staining intensity for both cGAS and PD-L1 is noticeably reduced in the TOP1 knockdown samples when compared to the control *in vivo*. Scale bars = 50 μm. **(H)** cGAS expression in cryosections from xenograft tumors was evaluated by confocal microscopy. All results are representative of observation from three biological replicates. Scale bars = 10 μm. **(I)** The concentrations of IFN-β were measured using ELISA *in vivo* (n = 5 mice/group). All results are representative of observation shown as means ± SD. The significance is indicated as N.S.

Considering the suggested interaction between TOP1 and cGAS, we conducted further investigations to determine whether TOP1 regulates cGAS in CC and its consequent effects. The expression levels of cGAS and its downstream proteins, such as NF-κB and IRF3, were determined using Western blot in TOP1 knockdown cells. Figure 5A and B show that inhibition of TOP1 resulted in impaired expression of cGAS, STING, NF-κB, and IRF3. The loss of cGAS impeded the production of the second messenger 2′-3′-cGAMP (Figure 5C). However, we did not observe phosphorylated IRF3. Consequently, IRF3-dependent IFNβ production was not altered in the knockdown cells (Figure 5D), while Poly (I:C) (polyinosinic: polycytidylic acid) was included as a positive control to induce IFNβ production (Field et al., 2010). Instead, we observed downregulation of CD274 (PD-L1) expression following TOP1 knockdown (Figures 5A, B), which can be triggered by cGAS activation as previously reported (Sato et al., 2019; Wang et al., 2023). The correlation between CD274 and cGAS in CC was demonstrated using TIMER databases (Figure 5E). The production of PD-L1 but not IFNβ suggests that TOP1 triggers cGAS activation independent of the canonical cGAS–IRF3-IFNβ cascade. Moreover, the reduction in staining intensity of cGAS and PD-L1 *in vivo* tissue samples from TOP1 knockdown model is consistent with our observed *in vitro* experiments, as depicted in Figure 5G. Vivo studies using tumor-bearing mice also showed no impact of TOP1 knockdown on serum IFNβ levels, which corroborate our findings in CC cells (Figure 5I), despite significant inhibition of cGAS expression by TOP1 knockdown (Figure 5H).

**FIGURE 6:**
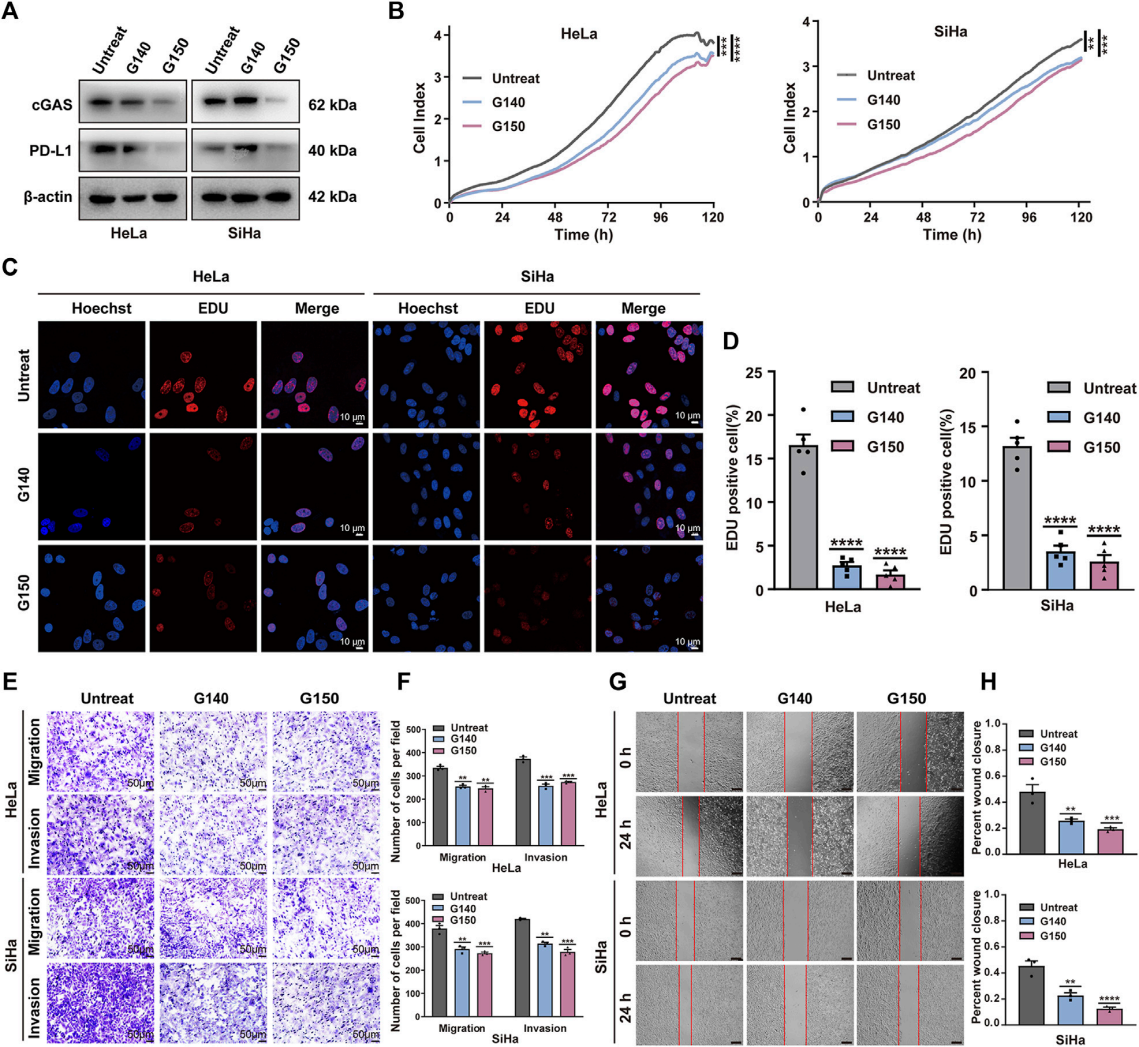
Effect of expression of cGAS on malignant behavior of CC cell *in vitro*. **(A)** The expression levels of cGAS and PD-L1 was confirmed using Western blot treated with or without cGAS inhibitor G140 (5 μM, 24 h) and G150 (5 μM, 24 h) in CC cells. **(B)** Cell proliferation of CC cells treated with cGAS inhibitors was assessed using RTCA assay within 5 days. **(C,D)** Proliferation analysis based on EdU incorporation in CC cells with or without cGAS inhibitors. The quantification of EdU positive cells was performed using ImageJ software. **(E,F)** The transwell assay showed inhibition of cGAS suppressed tumor cell migration and invasion processes. The results are presented in bar graphs for simplicity. Scale bar = 50 μm. **(G,H)** Wound healing assay demonstrated that inhibition of cGAS reduced tumor cell migration, illustrated as the bar Figures. Scale bar = 100 μm **P < 0.01; ***P < 0.001; ****P < 0.0001.

To test whether TOP1-dependent PD-L1 production relies on cGAS, we compensated for TOP1 knockdown cells with cGAMP (5 μM, 12 h), mimicking cGAS activation. The result showed a significant rescue of PD-L1 inhibition caused by TOP1 knockdown. In contrast, overexpression of TBK1 or IRF3 in the knockdown cells did not increase PD-L1 levels (Figure 5F). Moreover, the expression of PD-L1 could be significantly inhibited by cGAS-specific small molecule inhibitors G140 and G150 (Figure 6A). Interestingly, targeting cGAS also led to significant inhibition of CC cells’ proliferation, migration, and invasion (Figures 6B–H), confirming the involvement of cGAS in the development of CC. Taken together, our data show that TOP1 regulates PD-L1 production via activation of the non-canonical cGAS pathway.

### 3.5 Recognition of TOP1-cGAS interaction in CC cells

To determine if TOP1-dependent cGAS activation occurs through interaction, we conducted Co-IP assays in CC cells, both endogenously and exogenously. Supplementary Figure S2A illustrates a complex comprising endogenous TOP1 and cGAS in CC cells. Alternatively, Flag-TOP1 and HA-cGAS were co-transfected into CC cells and subjected to Co-IP as-says to further confirm the interaction (Supplementary Figure S2B). Moreover, immunofluorescent microscopy was used to determine the co-localization of TOP1 with cGAS. TOP1 was observed in the nucleus, while cGAS was predominantly found in the nucleus but also had a weaker presence in the cytosol (Supplementary Figure S2C). The corresponding fluorescent in-tensity profiles are shown in Supplementary Figure S2D. Additionally, our findings were supported by correlation analysis utilizing the Tumor Immune Estimation Resource (TIMER) database (Supplementary Figure S2E).

### 3.6 HPV oncoprotein E6/E7 are required for TOP1 induction and PD-L1 production

**FIGURE 7:**
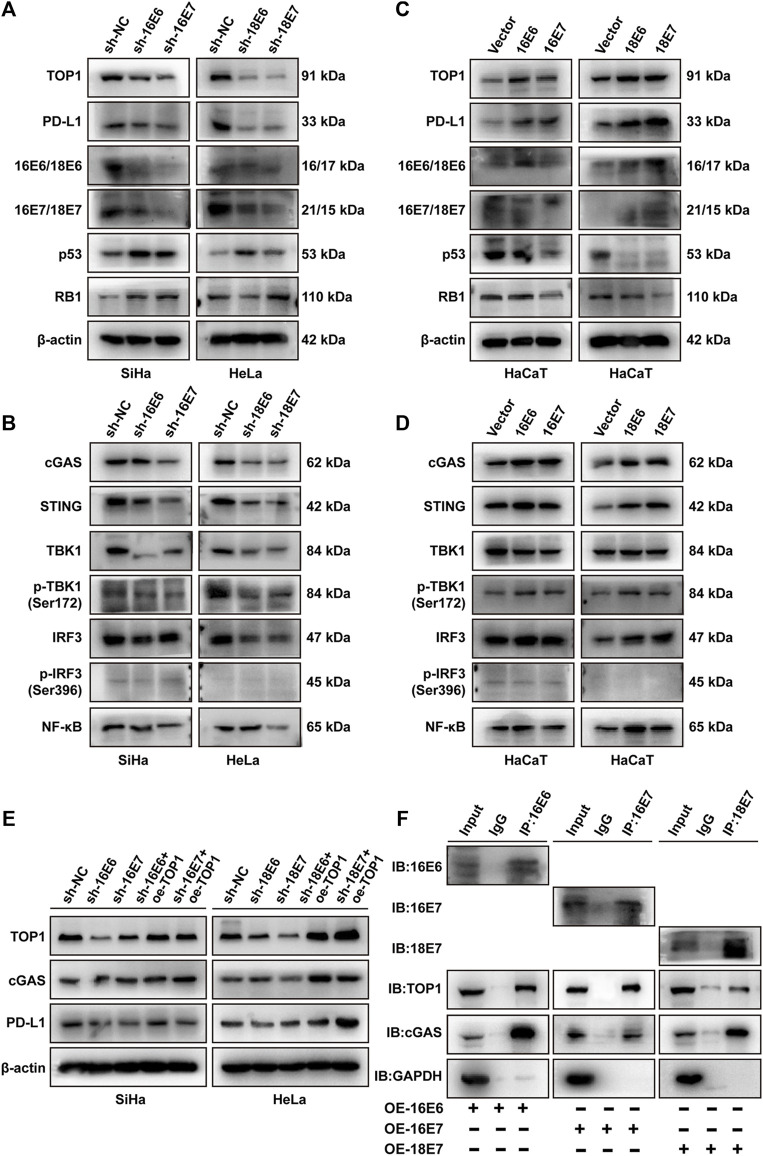
E6/E7 promotes the activation of TOP1-dependent cGAS-PD-L1 pathway. **(A,B)** Western blot analysis of TOP1, PD-L1, and related proteins of cGAS pathway in SiHa or HeLa cells transfected with sh16-E6/E7 or sh18-E6/E7. **(C,D)** Western blot detection of the indicated protein levels of TOP1, PD-L1, and related proteins of cGAS pathway in HPV16 E6/E7-and HPV18 E6/E7-overexpressing HaCaT cells. **(E)** Western blot analysis against the indicated proteins in shHPV16 E6/E7-expressing SiHa and shHPV18 E6/E7-expressing HeLa cells transfected with Flag-TOP1. **(F)** The Co-IP assay is performed using E6/E7 antibodies to detect the interaction between E6/E7 and TOP1 or cGAS in E6/E7-overexpressing cells, visualized using IB. GAPDH was used as a loading control. IP: immunoprecipitation; IB: immunoblotting.

To elucidate the mechanisms underlying the upregulation of TOP1 in CC, we examined the impact of oncoproteins E6 and E7 on TOP1 expression and PD-L1 production. Specific shRNAs targeting HPV16 E6/E7 or HPV18 E6/E7 were used to suppress their expression in CC cells. Western blot analysis revealed that the shRNAs effectively reduced the expression of E6 or E7, but E6 shRNAs could not bypass E7 due to their shared common nucleotide sequences. As a control, increased levels of p53 and RB1 were induced by the inhibition of E6/E7. Suppressing either E6 or E7 led to decreased expression of TOP1 and PD-L1, with E7 exhibiting greater efficiency (Figure 7A). Consistently, the levels of cGAS, STING, TBK1, IRF3, and NF-κB proteins or phosphor-proteins were decreased in the transduced cells (Figure 7B). Notably, p-IRF3 levels were low in both the control and transduced cells, suggesting that CC cells inhibit the activation of IRF3 regardless of E6/E7 suppression.

We further transiently overexpressed HPV16 E6/E7 or HPV18 E6/E7 in HPV-negative HaCaT cells. As shown in Figure 7C, both E6 and E7 upregulated TOP1 and PD-L1. E6 overexpression significantly induced downregulation of p53, while E7 overexpression modestly reduced RB1 levels. In contrast with shRNA studies, HPV16 or HPV18 oncoprotein expression resulted in the activation of the cGAS pathway (Figure 7D). Additionally, we performed rescue experiments in E6/E7 silencing cells. The overexpression of TOP1 partially restored the expression of cGAS and PD-L1 (Figure 7E), suggesting the critical roles of TOP1 in E6/E7-dependent PD-L1 production. Considering the interaction between TOP1 and cGAS demonstrated in Supplementary Figure S2, we further conducted Co-IP experiments to determine whether E6 or E7 interacted with the TOP1/cGAS complex. Figure 7F confirmed that E6 or E7 is individually capable of interacting with TOP1 and cGAS. Taken together, E6/E7 function as upstream regulators of TOP1, facilitating the activation of the cGAS-PD-L1 pathway.

### 3.7 Other downstream targets of TOP1 in CC cells

**FIGURE 8:**
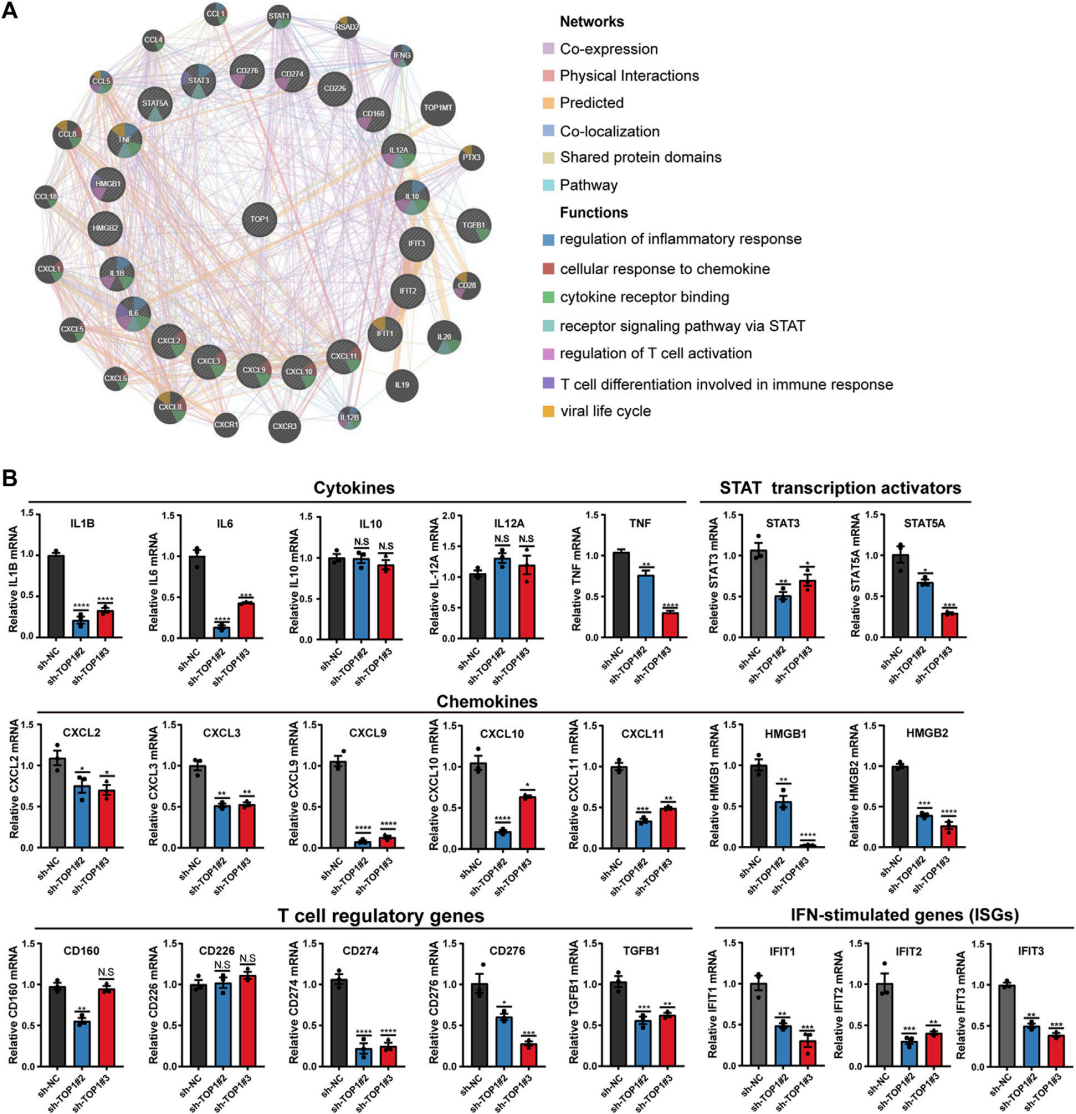
TOP1 inhibition suppresses expression of tumor-promoting inflammatory genes. **(A)** Protein-protein interaction (PPI) network by Gene MANIA predicts the networks and functions of TOP1 and gene sets. **(B)** qRT-PCR analysis of the indicated genes in TOP1 knockdown cells. The data represents the mean ± SD from three biological samples, Student t test is applied to detect statistical significance. *P < 0.05, **P < 0.01, ***P < 0.001, and ****P < 0.0001.

To comprehensively analyze TOP1-dependent candidates in CC development, The functional interaction network of TOP1 with immune-related genes was visualized using the GeneMANIA database, revealing that TOP1 also impacts other functions such as the regulation of inflammatory response, T cell activation, differentiation as well as viral life cycle (Figure 8A). To validate the expression of identified downstream targets, qRT-PCR analysis was conducted using TOP1 knockdown HeLa cells. The results demonstrated reduced mRNA levels of immune-related genes, including certain cytokines (IL-1B, IL-6, TNF), chemokines (CXCL2, CXCL3, CXCL9, CXCL10, CXCL11, HMGB1, HMGB2), STAT transcription activators (STAT3, STAT5A), T cell regulatory genes (CD274, CD276, TGFB1), and multiple interferon-stimulated genes (IFIT1, 2, 3) (Figure 8B). Among these, IL-1B, IL-6, TNF, CXCLs, TGFB1, HMGB1, STAT3, and STAT5 have been reported to contribute to tumor formation. Notably, the mRNA levels of IL-6 and IL-1B, rather than IL-10 and IL-12A, were impacted by TOP1. The expression of high mobility group box (HMGB) proteins, known as chemokines that bind to DNA to regulate transcription or immunological tolerance (Kazama et al., 2008; Chen et al., 2021; Voong et al., 2021), was significantly altered by TOP1 silencing. Additionally, the levels of STAT3 and STAT5A, as transcriptional regulators of cell proliferation and differentiation (Mui, 1999), were positively regulated by TOP1 in CC cells. Interferon-induced proteins with tetratricopeptide repeats (IFITs), particularly IFIT1 which interacts with HPV E1 protein (Terenzi et al., 2008), were repressed by TOP1 knockdown. Furthermore, TOP1 was found to regulate several T cell regulatory genes including CD274 and CD276, which serve as markers of limiting T-cell effector response or anti-cancer immunity. In summary, these findings suggest that TOP1 plays a crucial role in regulating tumor-promoting inflammation as well as PD-L1 production in CC.

## 4 Discussion

**FIGURE 9:**
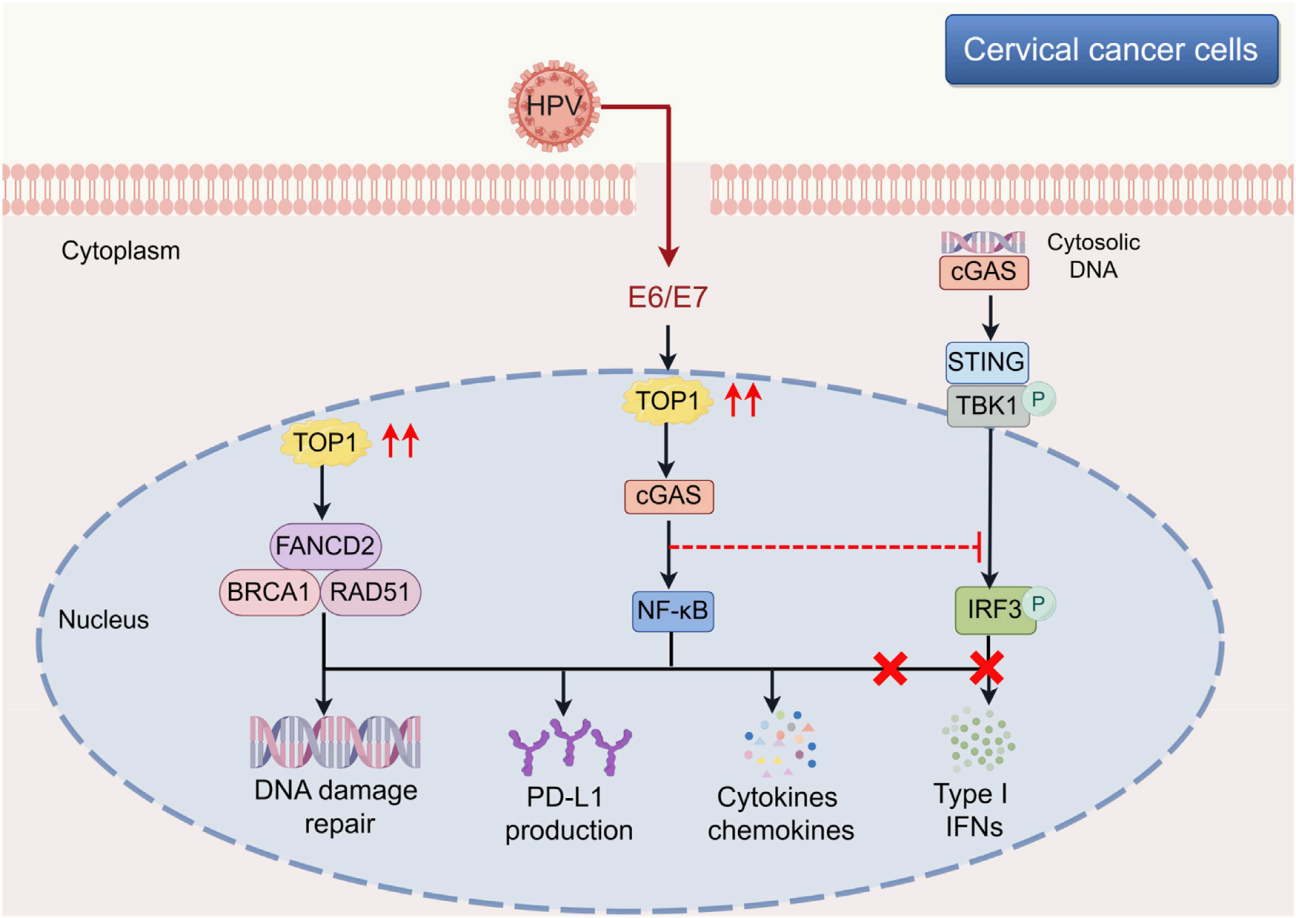
Involvement of TOP1-mediated cGAS-PD-L1 pathway during the CC development. The schematic illustration offers a comprehensive view of the molecular underpinnings within cervical cancer cells, with a particular emphasis on the influence of the TOP1-cGAS-PD-L1 pathway on tumor progression. TOP1’s multifaceted role is highlighted, showing its engagement in DNA repair mechanisms alongside FANCD2 and BRCA1-RAD51, which is essential for preserving genomic integrity amidst the genomic chaos of cancer. Furthermore, TOP1’s activation of cGAS in response to cytosolic DNA sets off a signaling cascade that, contrary to the typical induction of type I interferons, leads to the activation of the STING and NF-κB pathways, culminating in the upregulation of PD-L1 and other cytokines. The elucidation of the TOP1-cGAS-PD-L1 axis reveals it as a key target for novel therapeutic approaches. The schematic cartoon was created using Figdraw (https://www.figdraw.com). CC, cervical cancer; cGAS, cyclic GMP-AMP synthase; TOP1, topoisomerase I.

TOP1 has been identified as a crucial DDR protein involved in maintaining genome integrity. In this study, we unveil a novel non-DDR function of TOP1 in CC development. Elevated expression levels of TOP1 facilitate CC cell proliferation, migration, and invasion, while also impacting DDR in CC. Furthermore, TOP1 regulates cGAS, thereby inducing the expression of PD-L1 without affecting IFN-β expression in CC. Knockdown of TOP1 results in a significant reduction in cGAS, NF-κB, and PD-L1 levels. Notably, cGAS co-localizes with TOP1 in the nucleus, and pharmacological inhibition of cGAS suppresses CC cell growth. The upregulation of TOP1 in CC and its interaction with cGAS are primarily attributed to the essential oncoproteins E6 and E7. Additionally, we investigate the downstream effectors of TOP1 in CC cells and identify TOP1-mediated tumor-promoting inflammatory features, characterized by alterations in the expression of CXCLs, HMGB1, IL-6, and STAT3/5, as well as several T cell regulatory genes. A schematic representation of this process is illustrated in Figure 9.

Our findings regarding the interaction between TOP1 and cGAS are consistent with the previous report by (Zhao et al., 2020). However, Zhang et al. found that TOP1 inhibition induced a cGAS-dependent senescence-associated secretory phenotype (SASP), whereas we observed that inhibition of TOP1 in CC cells did not affect the expression of SASP-related factors such as IFN-β at the protein level (Figure 5D). This implies the existence of a secondary switch in CC cells that overrides the effect of TOP1 on IFN-β expression. Two plausible mechanisms may elucidate this phenomenon. On one hand, IRF3 may interact with E6 to suppress its transcriptional activities (Ronco et al., 1998). Consistently, undetectable p-IRF3 levels were observed in TOP1 knockdown cells (Figure 5A). In contrast, HPV18 E7 interacts with STING to antagonize IFN-β induction (Lou et al., 2023). It was also suggested that HPV has unique vesicular trafficking mechanisms to evade anti-tumor immunity (Uhlorn et al., 2020). Contrary to the suppression of SASP factors, we identified an increase in PD-L1 levels downstream of the TOP1/cGAS signaling pathway, regulated by oncoproteins (Figure 7C). Exogenous cGAMP treatment restores the loss of PD-L1 in TOP1 knockdown cells (Figure 5F). This upregulation suggests that TOP1 plays important roles in facilitating CC cell evasion of T cell surveillance. This regulatory effect of TOP1 on cGAS and PD-L1 *in vivo* reinforces the potential of TOP1 as a therapeutic target and illustrates the intricate interplay of cellular signaling in cancer progression. The clinical implications of our findings are significant, particularly regarding the potential synergy of TOP1 inhibitors (such as TPT or CPT) with checkpoint blockade therapies (such as anti-PD-L1 antibodies). This integrated therapeutic approach could be especially beneficial for CC patients exhibiting high TOP1 activity and PD-L1 expression, offering a novel avenue for more efficacious treatment strategies against CC.

As a recognized cytosolic DNA sensor that activates anti-tumor immunity, cGAS has been less studied in its relationship with HPV proteins compared to IRF3 or STING. Our study provides evidence that the interaction between TOP1 and cGAS accompanies the presence of HPV oncoproteins in CC cells (Figure 7F). Overexpression of E6 or E7 significantly increases the expression of cGAS (Figure 7D), whereas silencing E6 or E7 reduces the levels of cGAS (Figure 7B). Our observation is consistent with the findings of Laimin et al. that HPV E6 increases cGAS expression levels via p53 inhibition (Gusho and Laimins, 2022). Furthermore, we revealed that cGAS was predominantly localized in the nucleus (Supplementary Figure S2C) where TOP1 and E6/E7 are highly expressed. Zhang et al. demonstrated that TOP1 poisoning by CPT facilitated cGAS recognition of cytoplasmic chromatin, indicating that low expression of TOP1 allowed more cGAS to stay in the cytosol, which is consistent with our finding to some extent. It is undetermined whether TOP1 or HPV oncoproteins facilitate nuclear cGAS accumulation. Our findings raise another question regarding the function of nuclear cGAS in CC cells. Previous studies showed that phosphorylated cGAS in the nucleus suppressed HR-mediated DNA repair (Liu et al., 2018), yet we observed fewer DNA breaks in our cell models that expressed a high level of nuclear cGAS (Figure 3C).

In addition to investigating the TOP1-cGAS interaction, we further explored other potential downstream targets of TOP1. Our findings revealed that TOP1 regulates numerous genes with pro-cancer inflammatory features. Recent studies by the Marazzi group have reported that TOP1 inhibition protects against cell death induced by SARS-CoV-2 infection and suggested that TOP1 contributes to viral infection-induced pro-inflammatory responses (Rialdi et al., 2016; Ho et al., 2021). However, our results demonstrated that TOP1 knockdown suppressed CC cell growth *in vitro* (Figure 2), and consistent with this, *in vivo* studies showed that TOP1 inhibition dampens the formation of xenografted tumors (Figure 4B). These findings suggest that TOP1 does not induce a pro-inflammatory response against CC formation but potentially acts in opposing aspects to promote CC development. Marazzi et al. reported that TOP1 mediates pro-inflammatory genes induced by pathogen-associated molecular patterns (PAMPs), such as CXCL2/3/8, DDX60L, and OAS. The discovery that TOP1 regulates a range of cytokines and chemokines provides a foundation for developing combined therapeutic approaches for CC. By targeting TOP1 alongside specific inflammatory mediators, such as IL-6 or TNF, we may be able to disrupt the pro-inflammatory environment that supports CC growth. In contrast, our data demonstrate that TOP1 triggers a series of cytokines and chemokines, including HMGB1/2, CXCL9/10/11, IL-6, and TNF, as well as T cell regulatory genes such as TGFB1 and PD-L1 (Figure 8B). This difference might be attributed to the distinct functions of HPV compared to the RNA viruses studied by Marazzi et al. Additionally, there may be different outcomes in terms of inflammation between episomal and integrated forms of HPV. For instance, episomal HPV infection suppresses GATA4-dependent pro-inflammatory gene expression (Kang et al., 2015), whereas our research is associated with HPV integrated form. Comparative studies between episomal and integrated forms of HPV in relation to TOP1’s role in inflammation may provide insights into the heterogeneity of CC and inform treatment strategies.

## Data Availability

The original contributions presented in the study are included in the article/Supplementary Material, further inquiries can be directed to the corresponding authors.
