# Supplementary material for: Oncoproteins E6 and E7 upregulate topoisomerase I to activate the cGAS-PD-L1 pathway in cervical cancer development
Source: Front Pharmacol. 2024 Aug 2;15:1450875. doi: 10.3389/fphar.2024.1450875 (PMC11327024; doi:10.3389/fphar.2024.1450875)
Supplement: Supplementary file 7 [file DataSheet1.docx]

Supplementary Material

# Supplementary Figures


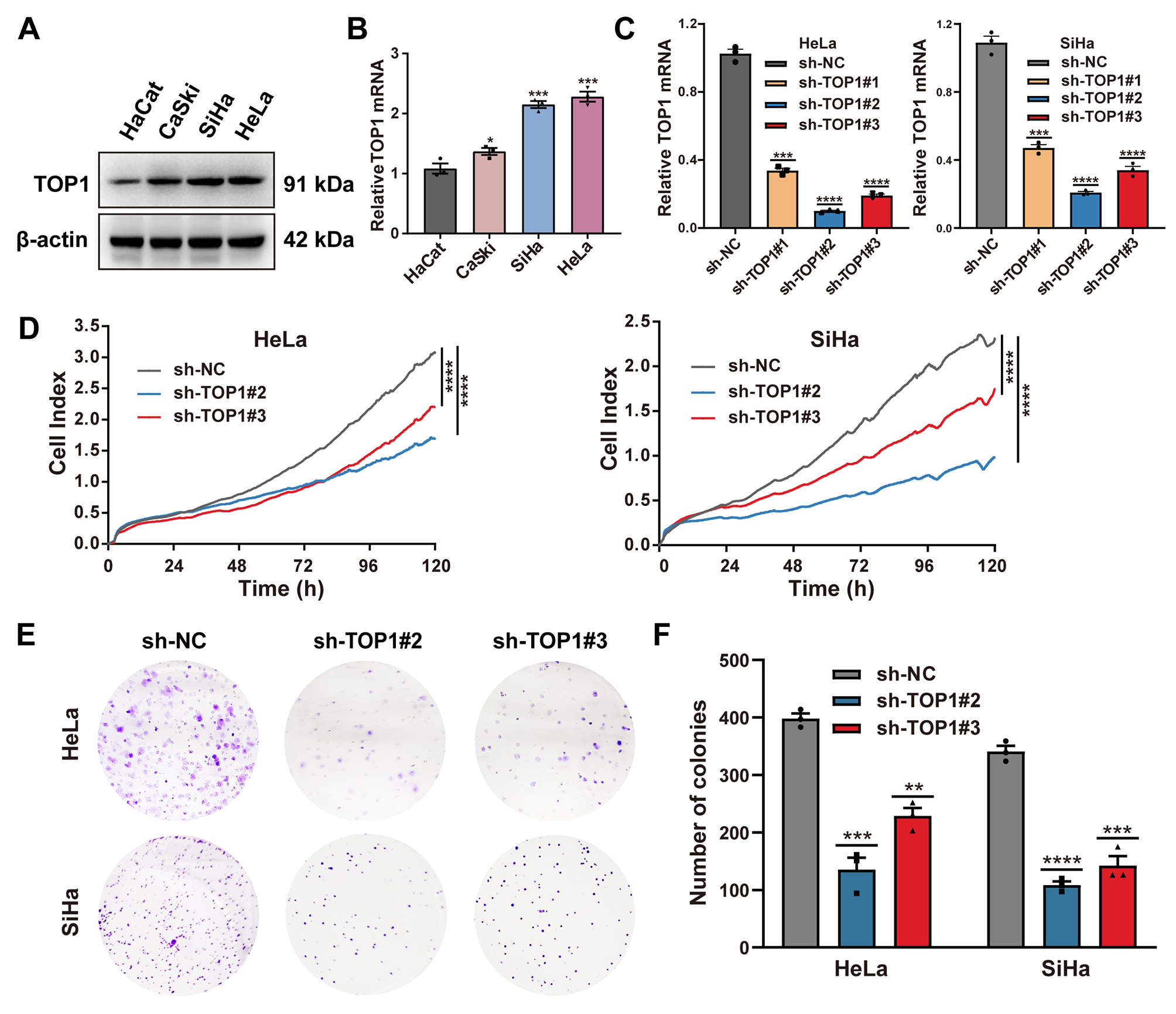


**Supplementary Figure S1.** TOP1 promotes cervical cancer expansion. **(A)** Western blot analysis of TOP1 protein level in HPV-negative (HaCaT) and positive (CaSki, SiHa, HeLa) cells. **(B)** qRT-PCR of TOP1 mRNA level in HPV negative and positive cells. **(C)** qRT-PCR of TOP1 mRNA in control and TOP1-knockdown (sh-TOP1#1, sh-TOP1#2, sh-TOP1#3) HeLa and SiHa cells. **(D)** RTCA assay illustrating proliferation of the control and TOP1 knockdown cells at 0, 24, 48,72, 96, 120 h. **(E)** Detection of colony formation imTOP1 silenced and control SiHa and HeLa cells. (F) The statistical quantification of colony formation assay (Each value represented as the mean ± SD for triplicate samples, Student t test). ***P*<0.01, ****P*<0.001, and *****P*<0.0001.


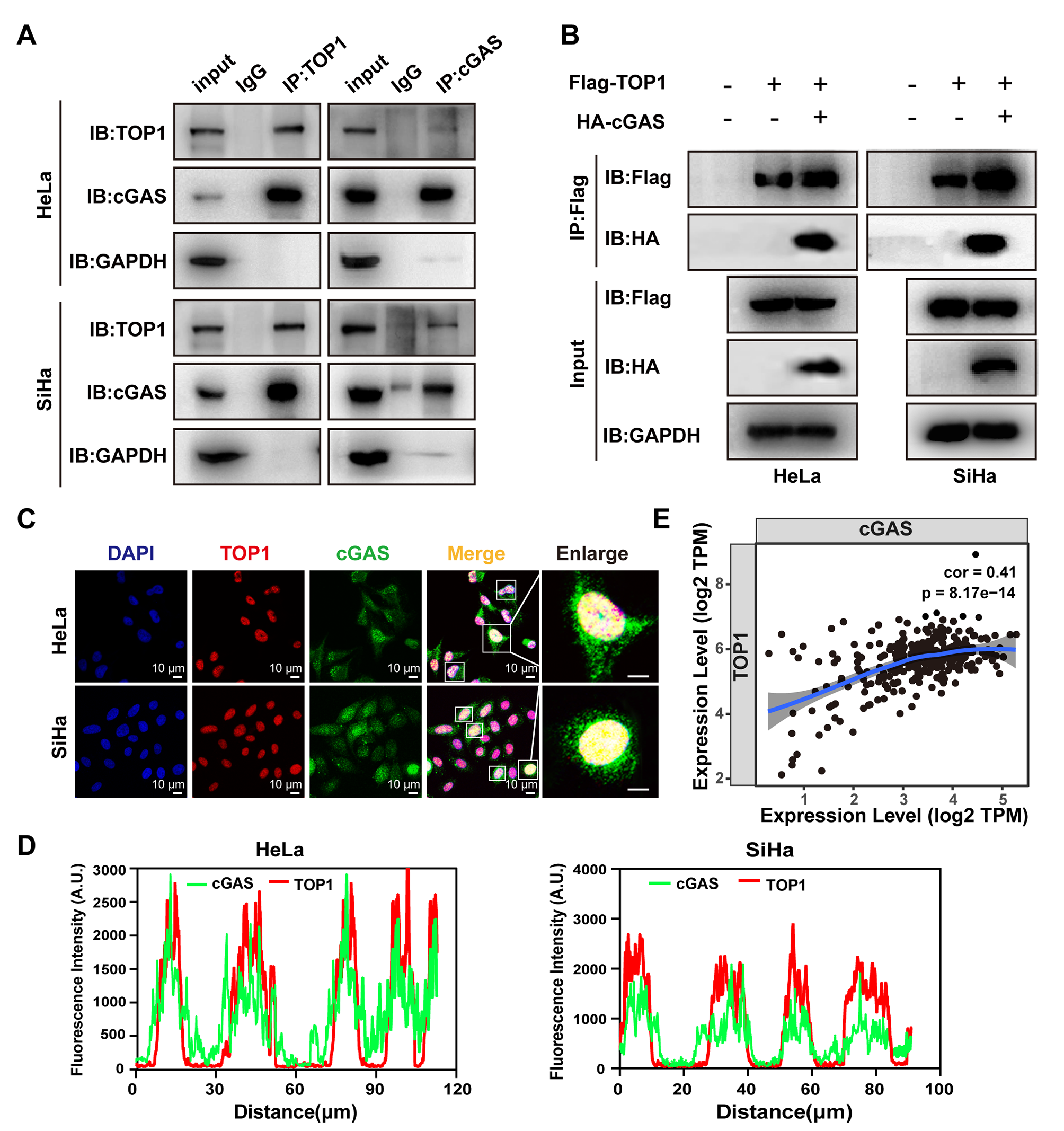


**Supplementary Figure S2.** Recognition of TOP1-cGAS interaction in CC cells. **(A)** Co-immunoprecipitation (Co-IP) assay was performed using CC cell lysates to detect the interaction of endogenous TOP1 and cGAS in SiHa and HeLa cells, GAPDH was used as endogenous control. **(B)** Co-IP analysis of the interaction between overexpressed Flag-TOP1 and HA-cGAS in CC cells. IP: immunoprecipitation; IB: immunoblotting. **(C)** Representative images of Immunofluorescence staining of cGAS (green) and TOP1 (red) in CC cells. The nuclei were shown DAPI (blue) staining. Scale bar=10 μm. **(D)** The interaction was confirmed by localization analysis using ImageJ software. **(E)** TIMER data-base-based correlation analysis between TOP1 and cGAS in CC specimens, together with the Spearman’s rho value (Cor=0.41) and estimated statistical significance (*P*=8.17e-14).

#
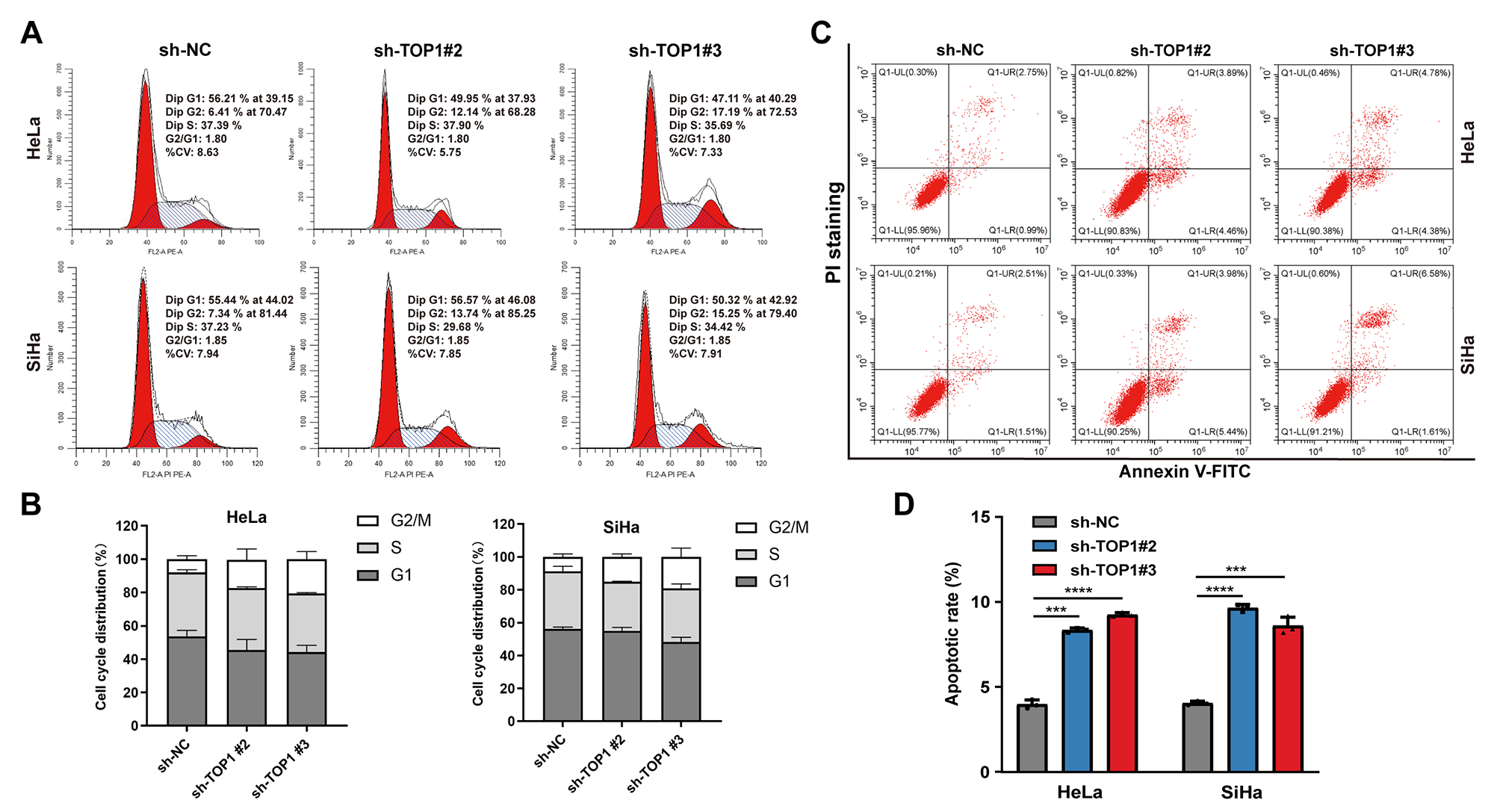


**Supplementary Figure S3.** Cell cycle disruption and enhanced apoptosis in TOP1 knockdown cells. **(A)** Cell cycle distribution of TOP1 knockdown cells as determined by flow cytometry. The data are presented as the number of effective cells counted on the Y-axis against the DNA content on the X-axis. The indicated peaks correspond to G1 (Dip G1), S (Dip S), and G2/M (Dip G2) phases, with the percentage of cells in each phase annotated**.** **(B)** The graph represents the cellular distribution analysis across different phases of the cell cycle, with specific percentages provided for G1, S, and G2/M phases. **(C)** Apoptosis analysis of TOP1 knockdown cells using Annexin V-FITC and propidium iodide (PI) staining. The plot shows the percentage of viable (lower left quadrant), early apoptotic (lower right quadrant), late apoptotic (upper right quadrant), and necrotic cells (upper left quadrant). The graph is divided into four quadrants, each representing different stages of cell death. **(D)** This graph presents quantification analysis of apoptotic cell populations based on fluorescence intensity.

Immunofluorescence, Immunohistochemistry staining images and Western Blot images are upload to jianguoyun. Link as follows

File 1. Immunofluorescence staining images <https://www.jianguoyun.com/p/DeiWqg8Qr-bYDBihlsoFIAA>

File 2. Immunohistochemistry and HE images <https://www.jianguoyun.com/p/Dd-N3HUQr-bYDBiilsoFIAA>

File 3. Western Blot images <https://www.jianguoyun.com/p/DTfEyhcQr-bYDBillsoFIAA>

# Supplementary Tables are displayed in EXCEL format, including Table S1-S6.
